# Supplementary material for: Masked face matching benefits from isolated facial features
Source: PLoS One. 2025 Jul 1;20(7):e0326706. doi: 10.1371/journal.pone.0326706 (PMC12212515; doi:10.1371/journal.pone.0326706)
Supplement: Attachment — Submitted filename: Response to reviewers.docx [file pone.0326706.s002.docx]

Dear Dr. Ptaszynski,

Thank you for the opportunity to revise our manuscript and the valuable feedback from you and the reviewers.

We have carefully considered all comments and suggestions and have revised the manuscript to meet the requirements of *PLoS ONE*. Below is a detailed point-by-point response to each of yours and the reviewers' comments. Changes made to the manuscript are highlighted in red for your reference.

**Editor’s comments**

1. **Please ensure that your manuscript meets PLOS ONE's style requirements, including those for file naming.**

Thank you for your feedback. We have carefully revised our manuscript to ensure that it fully complies with PLoS ONE's style requirements.

2. **Thank you for stating the following financial disclosure:**

**China Scholarships Council, file NO. 202108210121. Please state what role the funders took in the study. If the funders had no role, please state: "The funders had no role in study design, data collection and analysis, decision to publish, or preparation of the manuscript." If this statement is not correct you must amend it as needed. Please include this amended Role of Funder statement in your cover letter; we will change the online submission form on your behalf. Please remove any funding-related text from the manuscript and let us know how you would like to update your Funding Statement. Currently, your Funding Statement reads as follows: China Scholarships Council, file NO. 202108210121.**

**We note that you have provided funding information that is not currently declared in your Funding Statement. However, funding information should not appear in the Acknowledgments section or other areas of your manuscript. We will only publish funding information present in the Funding Statement section of the online submission form. Please include your amended statements within your cover letter; we will change the online submission form on your behalf.**

Thank you for your guidance regarding the funding statement. We have removed all funding-related text from the manuscript and have included the following amended Role of Funder statement in our cover letter: "*The funders had no role in study design, data collection and analysis, decision to publish, or preparation of the manuscript.*"

Given this change, we would appreciate if you could update the Funding Statement in the submission system as follows: *Funding Statement: This work was supported by a studentship from the China Scholarships Council (File No. 202108210121).*

3. **Your abstract cannot contain citations. Please only include citations in the body text of the manuscript, and ensure that they remain in ascending numerical order on first mention.**

We reviewed our abstract and confirm that it does not contain any citations (see p. 2).

4. **We note that Figure 1 and 2 includes an image of a [patient / participant / in the study].
   As per the PLOS ONE policy (http://journals.plos.org/plosone/s/submission-guidelines#loc-human-subjects-research) on papers that include identifying, or potentially identifying, information, the individual(s) or parent(s)/guardian(s) must be informed of the terms of the PLOS open-access (CC-BY) license and provide specific permission for publication of these details under the terms of this license. Please download the Consent Form for Publication in a PLOS Journal (http://journals.plos.org/plosone/s/file?id=8ce6/plos-consent-form-english.pdf). The signed consent form should not be submitted with the manuscript, but should be securely filed in the individual's case notes. Please amend the methods section and ethics statement of the manuscript to explicitly state that the patient/participant has provided consent for publication: “The individual in this manuscript has given written informed consent (as outlined in PLOS consent form) to publish these case details”.**

**If you are unable to obtain consent from the subject of the photograph, you will need to remove the figure and any other textual identifying information or case descriptions for this individual.**

We have removed the figures related to the facial image dataset from the manuscript. Accordingly, the revised version no longer contains any images of individuals. We believe this revision fully satisfies the journal’s requirements regarding informed consent and the publication of potentially identifiable information.

5. **We note you have included a table to which you do not refer in the text of your manuscript. Please ensure that you refer to Table 1 in your text; if accepted, production will need this reference to link the reader to the Table.**

Thanks for pointing this out, we have now added a reference to Table 1 in the main text: *“Table 1 presents a summary of all hypotheses”* (see p. 6).

6. **Please include captions for your Supporting Information files at the end of your manuscript, and update any in-text citations to match accordingly. Please see our Supporting Information guidelines for more information:** [**http://journals.plos.org/plosone/s/supporting-information**](http://journals.plos.org/plosone/s/supporting-information)**.**

In response to the second reviewer’s comments, we moved the gender difference analysis from the supplementary to the main text. As a result, the manuscript no longer has a Supporting Information section.

**Reviewer 1 comments**

1. **The title should precisely declare which research question is solved, but the title of the manuscript is general. It is recommended to clearly categorize the studied work and present the research aim in a precise title. For example, write the exact problem-solving of the research in the title.**

We appreciate the reviewer’s suggestion regarding the title. Our study aims to examine the effectiveness of presenting isolated features, such as the eyes (Experiment 1) and the mouth (Experiment 2) in enhancing face-matching accuracy for masked targets compared to using the full face. The title clearly states the aim/“problem”: *“Improving Identity Matching for Masked Faces”* and the method/“problem solving” we used to achieve this “*The Benefit of Isolated Facial Features”*. However, to enhance precision and address the reviewer’s suggestion, we have revised the title to: *"**Masked Face Matching Benefits from Isolated Facial Features."*

2. **Abstract is too vague; it contains some sentences but some important aspects of abstract are missing. It should contain problem, challenges, method and evaluation clearly. It is recommended to rewrite the abstract.**

Based on your suggestions, we have revised the abstract to make the introduction of the problem and challenges clearer. Specifically, we have changed the opening to the following: “*Verifying the identity of an unfamiliar person is a difficult task, especially when targets wear masks that cover most of their faces. This presents a major challenge for law enforcement in border control, security, and criminal investigations. Therefore, we aim to explore ways to improve face matching performance when a face is heavily maske**d”.* (see p. 2)

Additionally, we have clarified the evaluation method now stating that: “*we investigated whether matching isolated facial features, namely the eyes (Experiment 1) and the mouth (Experiment 2), instead of a full face can improve matching accuracy when a target is masked”*.

We believe that the methods are presented in a clear way that aligns well with the expectations of psychological journals. Specifically, we describe the stimuli presented and the task of the participants in each condition: *“In congruent pairs, participants matched a full-face image to another full-face image or a masked image to an isolated feature. In incongruent pairs, participants matched a full-face image to an image of the eyes or the mouth only or to a masked image.*

Finally, we have simplified the sentence structure of the concluding sentence of the abstract to further improve the clarity of the proposed solution to the challenge: *“Overall, the two experiments showed that focusing on isolated facial features, such as the eyes or mouth, can be a valuable strategy for enhancing identity matching when dealing with masked perpetrators”.*

3. **Masked Face identity is introduced in the manuscript but some aspects of facial appearance that is affected on Facial identity matching are missing. For instance, it can be introduced aging or other appearances. Therefore contemplate "Toward E-appearance of human face and hair by age, expression and rejuvenation," 2004 International Conference on Cyberworlds, Tokyo, Japan, 2004, pp. 306-311, doi: 10.1109/CW.2004.65. “ then discuss about different appearances.**

We fully acknowledge that factors, such as aging, facial expressions, and other appearance-related changes can indeed affect facial identity matching. However, our work specifically examines the effect of full-face coverings that conceal most facial features, leaving only the eyes and mouth exposed. This setup effectively minimizes the influence of other facial features. Additionally, our work focuses on improving human face-matching performance when masks heavily obscure faces. Although exploring how systems predict appearances behind masks might enhance face matching is an intriguing research direction, it is beyond the scope of our current study.

4. **Use Natural masked face dataset. If input data has a face mask with the natural looking of human face how it works?**

We agree that exploring identity matching with more natural-looking masked faces is an important direction for future research. We acknowledge this point in the limitations section of the original submission: “*We used laboratory-based face-matching materials that had an idealized masked condition (clear, front face), which closely resembles the standardized photographs found in passports or other official identification documents. However, this differs from masked targets in real-life scenarios. In real life, face matching is even more challenging because of the poor quality of the images [77] and the passage of time.*” (p. 31)

However, the primary goal of the present study was to test our hypothesis in a controlled laboratory setting, ensuring internal validity and isolating the role of congruency and feature-based processing in masked face recognition. This controlled approach allowed us to establish a clear causal link between these factors and recognition performance without the additional confounds present in real-world data. Establishing this foundation is essential and necessary before extending the findings to more complex, real-world conditions.

While we have since conducted a separate study that examines masked face recognition using real-world images from various angles (see link: <https://osf.io/wqvr4/?view_only=f2453783d8444a979cf0dc476c6d2355>), that investigation adopts a different methodological framework and addresses distinct research questions that go beyond the scope of the present study. The current paper is focused on establishing the fundamental mechanisms underlying congruency effects in a controlled environment, which is a necessary step before extending these findings to more complex, real-world scenarios.

Regarding naturally masked faces, prior studies such as those of Manley et al. (2019) and Sagana & Hildebrandt (2022) have used stimuli where target faces wore masks. Their findings are comparable to ours, suggesting that seeing the encoded feature in isolation can enhance identification accuracy.

5. **The comparative study is not complete. Please make a comparative study.**

The comparative study is inherent in the experimental design. Our study systematically compares different approaches through distinct experimental conditions. Specifically, for masked targets, we compared the current standard practice—the benchmark model (masked-full face condition)—with an alternative approach that leverages feature-based processing (masked-partial condition). This comparison revealed a statistically significant improvement in face matching, amounting to a 13% increase in accuracy. Given that our aim was to isolate and assess the impact of feature-based processing in masked face recognition, the comparative aspect of our study is complete and directly addresses the research question.

Furthermore, we extensively compare our findings with prior studies in multiple ways:

1. Comparison with previous research: The literature on the topics we study in this manuscript is vast. Specifically , we refer to relevant studies on masked face recognition, e.g., “*Previous studies have indeed explored issues related to masked face recognition [7,8,21,23,24], however, most of them have focused on surgical masks, which obscure only a small portion of the face, leaving a large part of the face still visible. In contrast, full face coverings, such as those used by perpetrators, often leave only minimal portions of the face visible, such as the eyes*”. (see p. 5).
On the confidence-accuracy relationship, we refer to: “*The few existing studies suggest that in face matching, confidence is a good indicator of accuracy for both positive (“same person”) and negative (“different people”) decisions, when the proportion of matched and mismatched pairs in the task is equal [44,45]. However, to the best of our knowledge, no study to date has examined the confidence-accuracy relationship for face matching when the targets are masked.*” (see p. 7)

2. Comparison with previous results: we discussed how our results align with or differ from previous studies (see p. 28 - 31), focusing on various aspects:

a.	Congruency effect: *“ These findings are in line with previous work [7,8,30,66] and transfer appropriateness [22]. Importantly, unlike previous studies, we have shown that the effect persists even when the number of facial features to be matched is severely restricted. These findings provide strong support for the benefits of featural processing for improving face matching for masked targets [5,66]”.*

b.	Response bias: *“This conservative bias aligns well with findings showing that face masks lead to “mismatch” responses [7,28,66]”.*

c. Confidence-accuracy relationship: *“The lack of a clear confidence-accuracy relationship in our studies stands in stark contrast to the literature on the confidence-accuracy relationship for face recognition [69], lineups [43,48,67] and face-matching [44]”.*

d. Gender differences: *“the effect is inconsistent across experiments and the direction of the effect is inconsistent with previous studies [63-65]”.*

Finally, we also compare the results of Study 1 and Study 2, which examine different facial features, focusing on various aspects:

a. Congruency effect: “*Matching accuracy and discriminability were markedly higher for congruent than incongruent face pairs in both experiments, generating large effect sizes (η_p_^2^ ≥ .62).”* (p. 28)
b. Response bias: “*This conservative bias persisted in Experiment 2.”* (p. 29)
c. Confidence-accuracy relationship: “*we did not find the typical confidence-accuracy relationship in both experiments [41,43,67,68].*”(p. 30)
d. Decision time - accuracy relationship: “*decision times did not vary as a function of accuracy in Experiment 1 and Experiment 2.”* (p. 31)

This additional comparison further strengthens the comparative study of the present work, strengthening the theoretical and practical contribution of our work.

6. **The novel contributions are thus not clarified. Please explain where is the novelty of the proposed method.**

This line of research is aimed at improving face-matching performance in cases involving heavily occluded faces. This is relevant in criminal contexts but largely overlooked in prior studies. While most masked face matching and recognition research focused on pandemic-related surgical masks, we address the more challenging case of full-face coverings that obscure most facial features. Following your suggestion, we have clarified this novel contribution in the main text: “*Previous studies have indeed explored issues related to masked face recognition [7,8], however, most of them have focused on surgical masks, which obscure only a small portion of the face, leaving the large part of face still visible. In contrast, full face coverings, such as those used by perpetrators, often leave only minimal portions of the face visible, such as the eyes. These more substantial masks present a greater challenge for recognition, as they conceal a larger number of diagnostic features [23,24]”.* (see p. 5)

Novelty also comes from our proposal to leverage the use of feature-based processing, that is, focusing on isolated facial features when only limited portions of the face are exposed: “*In such cases, relying on isolated facial features rather than holistic processing may enhance matching performance, particularly when only limited portions of the face are available [14,19].”* (see p. 5). This is further emphasized in our research aims: *“The aims of the current line of research are to investigate the advantage of displaying isolated facial features for matching masked faces and to test whether this advantage holds for masked targets that reveal either their eyes or their mouth under extreme occlusion.”* (see p. 6)

Additionally, although confidence and decision time are well-studied accuracy indicators in lineup tasks (Sporer et al., 1995; Weber & Brewer, 2006; Wixted & Wells, 2017), their role in face matching, especially with masked face, remains unexplored. Our study fills this gap by analyzing their relationship to accuracy in masked face matching, contributing to a deeper understanding of meta-cognition in face processing under occlusion. We have clarified this novel contribution in our manuscript: “*to the best of our knowledge, no study to date has examined the confidence-accuracy relationship for face matching when the targets were masked”*, and “*To date, no study has investigated the relationship between decision time and accuracy in face matching.”* (see p. 8).

We believe our work offers novel key insights into improving face matching in heavily occluded scenarios, with practical applications in security and law enforcement.

7. **How much the proposed study is robust for the face with cosmetics?
   Consider “Toward anthropometric simulation of face rejuvenation and skin cosmetic,**<https://doi.org/10.1002/cav.38>**" and then show how the proposed study is robust for real applications?**

The study you referenced differs from ours in several key aspects. Our studies primarily address the challenge of identifying individuals whose faces are largely covered, which is highly relevant to real-world applications such as border control and law enforcement. The issues of cosmetics and skin tone are not central to our study. Specifically, features like the lips, cheeks, and skin tone—typically influenced by cosmetics—are hidden when part of the face is covered, which minimizes the impact of cosmetics on our task. Additionally, our study uses gray-scale facial images rather than color images (see p. 11). In gray-scale images, subtle variations in skin tone or the effects of cosmetic products, which would be visible in color, are less pronounced. This further reduces the influence of cosmetic factors on face matching, as the focus is primarily on the visible features of the face.

It is also important to note that in real-life applications, such as passport control or security checks, officers often rely on standardized photographs, like those found in passports or driver's licenses, which resemble the images in our studies. Therefore, our work reflects real-world challenges closely, where the focus is on identifying individuals despite face coverings or partial occlusion, we added this explanation in the main text: *“We used laboratory-based face-matching materials that had an idealized masked condition (clear, front face), which closely resembles the standardized photographs found in passports or other official identification documents. However, this differs from masked targets in real-life scenarios. In real life, face matching is even more challenging because of the poor quality of the images [77] and the passage of time”.* (see p. 31)

8. **The aim of the proposed method is not adequately explained. It appears that the research question has been previously addressed before.**

We agree that it is important to clearly state the aims. Therefore, we made it a point to formulate this aim in the abstract: “T*his presents a major challenge for law enforcement in border control, security, and criminal investigations. Therefore, we aim to explore ways to improve face matching performance when a face is heavily masked”* (see p. 2) and the last sentence of the first introduction paragraph in the initial submission (see p. 3), “*We aim to address this challenge of identifying masked perpetrators by investigating the effectiveness of matching isolated facial features”*.

For further clarification, we elaborate on this aim again by integrating existing studies before presenting the research hypotheses, *“The aims of the current line of research are to investigate the advantage of displaying isolated facial features for matching masked faces and to test whether this advantage holds for masked targets that reveal either their eyes or their mouth.” (see p. 6)*

As for the second part of the comment on the research question, please refer to our response to comment 6 above.

9. **The innovative aspect of the proposed method is not adequately explained.**

Please refer to our response to comment 6 above for a detailed explanation of the innovative aspect of the proposed method.

**Reviewer 2 comments**

1. **The authors put gender differences in a supplementary document, which is unnecessary. The complete part can be included in the main paper, unless there are some page limitations.**

In response to the comment, we agree to move the gender differences from the supplementary to the main text (see p. 13, p. 21, p. 27).

2. **I was hoping to see in the experiments, if possible, a comparison with auto-matching features not based on eyewitnesses and comparing to what extent the accuracy differs from the manual experiments.**

This work focuses on human face matching performance, particularly examining how isolated facial features benefit people from matching a masked perpetrator. The aim was to investigate human decision-making and meta-cognition, which are not directly applicable to automated face recognition systems.

While a comparison with auto-matching methods would indeed provide additional insights, it falls beyond the scope of the current line of research. However, we agree and acknowledge that it is important to consider auto-matching methods. Therefore, we added the following to our future direction (see p. 32): *“**Finally, because human face-matching decisions may be influenced by AI judgments [79], future research may integrate AI and human decision-making, particularly for masked faces, to enhance accuracy and reduce errors in border control systems, where AI alone may struggle with identity resolution [80]”.*
